# Supplementary material for: Transcriptomic Profiling of Breast Cancer Cells Induced by Tumor-Associated Macrophages Generates a Robust Prognostic Gene Signature
Source: Cancers (Basel). 2022 Oct 31;14(21):5364. doi: 10.3390/cancers14215364 (PMC9655582; doi:10.3390/cancers14215364)
Supplement: Supplementary file 1 [file cancers-14-05364-s001.zip › Supplementary Figure S1.pdf]

Supplementary Materials

Supplementary Tables

Supplementary Table1: Statistical analysis of differentially expressed genes in MCF7 transcriptome induced by MDMs or TAMs

Supplementary Table2: Statistical analysis of genes expressed between samples with 50 highest or lowest risk scores in TCGA-BRCA cohort.

Supplementary Table3: GO-GSEA analysis result based on the differentially expressed genes from Supplementary Table 2.

Supplementary Table4: Statistical analysis of estimated immune infiltration data between high- and low- riskscore groups in TCGA-BRCA cohort.

Supplementary Table5: Linear regression analysis result of the risk score and estimated drug IC50 values in TCGA-BRCA cohort.

Supplementary Figures and related methods

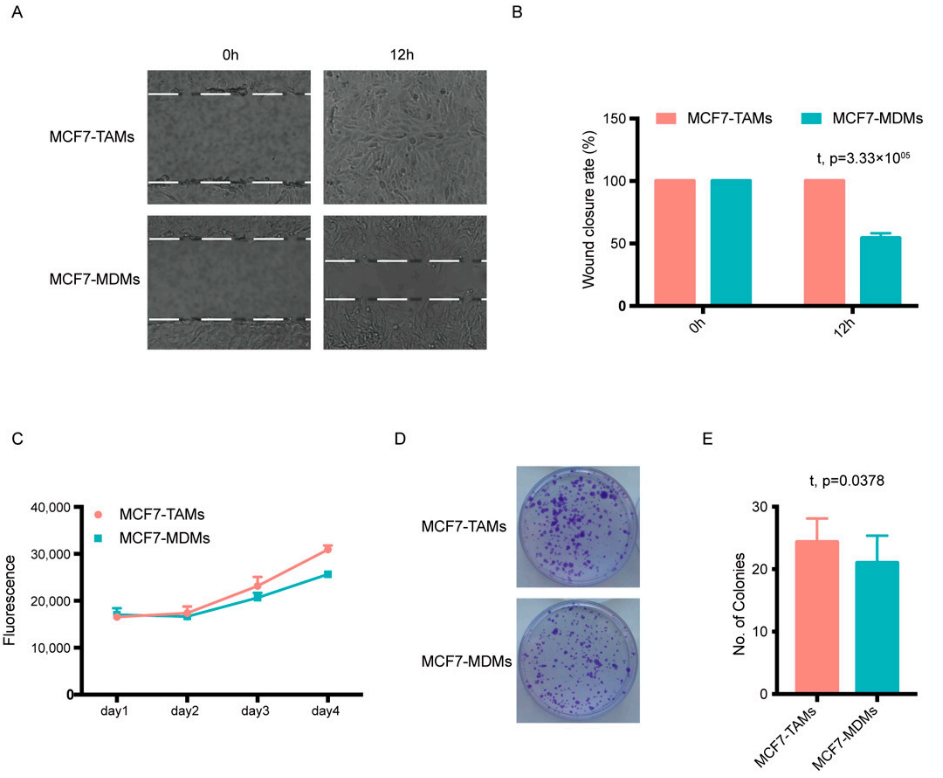

Supplementary Figure S1: Cell malignances induced by TAMs. (A) Representative photo of wound healing assay in MCF7 cells cultured in either TAMs CM or MDMs CM; (B) The wound closure rate in (A) was compared by t-test. Mean± s.d. was presented; (C) Cell proliferation of MCF7 cells cultured in either TAMs CM or MDMs CM was monitored by Alamar Blue Assay. Mean± s.d. was presented; (D) Representative photo of colony formation assay in MCF7 cells cultured in either TAMs CM or MDMs CM. (E) The number of colonies in (D) was compared by t-test. Mean± s.d. was presented.

Methods: AlamarBlue™ Cell Viability Reagent (DAL1100, Invitrogen) was employed and measured by Varioskan Flash multimode reader (Thermo Fisher Scientific) to monitor cell proliferation in 96-well plate at indicated time points. Three technical replicates were performed per assay. Briefly, the sample amount of MCF7 cells, cultured in TAMs CM or MDMs CM for 6 days, were plated in either 96-well plate for proliferation assay or p35 dish for colony formation assay. And the cells were maintained in the same media as before during the process. For wound healing assay, MCF7 cells cultured in TAMs CM or MDMs CM for 6 days were plated in 12-well plate. 24h later, the confluent monolayer was scratched using a p200 pipette tip and washed with PBS to remove the debris. The cells were cultured in TAMs CM or MDMs CM for another 12h and the migration area was assessed by the ratio of closure area to initial wound (t=0h) as follows: migration area (%)=(A0-A12)/A0*100, where A0 represents the area of initial wound area, A12 represents the residual area of wound 12h later.

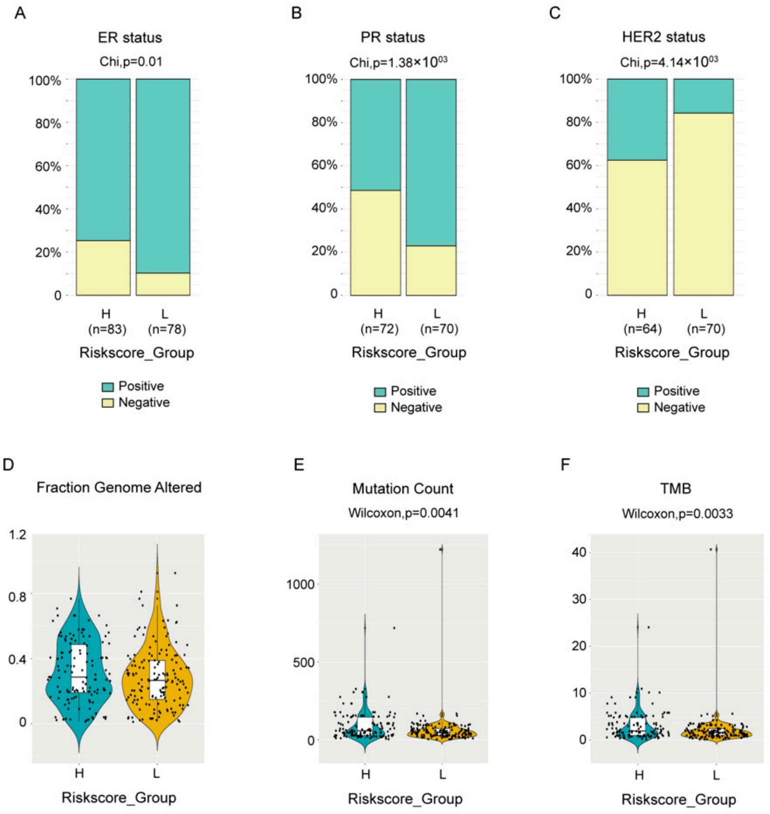

Supplementary Figure S2: Association of the risk score with clinical and biological features in MBC cohort. The difference in ER status (A), PR status (B), HER2 status (C), Fraction Genome Altered (D), Mutation Count (E) and TMB(F) between high- and low- riskscore groups in the MBC cohort.
